# The effectiveness of calcium electroporation combined with gene electrotransfer of a plasmid encoding IL-12 is tumor type-dependent

**DOI:** 10.3389/fimmu.2023.1189960

**Published:** 2023-05-25

**Authors:** Barbara Lisec, Bostjan Markelc, Katja Ursic Valentinuzzi, Gregor Sersa, Maja Cemazar

**Affiliations:** ^1^ Department of Experimental Oncology, Institute of Oncology Ljubljana, Ljubljana, Slovenia; ^2^ Faculty of Health Sciences, University of Ljubljana, Ljubljana, Slovenia; ^3^ Biotechnical Faculty, University of Ljubljana, Ljubljana, Slovenia; ^4^ Faculty of Health Sciences, University of Primorska, Izola, Slovenia

**Keywords:** calcium electroporation, electrochemotherapy (ECT), gene electrotransfer (GET), interleukin 12 (IL12), murine tumor models, bleomycin (BLM), calcium

## Abstract

**Introduction:**

In calcium electroporation (CaEP), electroporation enables the cellular uptake of supraphysiological concentrations of Ca^2+^, causing the induction of cell death. The effectiveness of CaEP has already been evaluated in clinical trials; however, confirmatory preclinical studies are still needed to further elucidate its effectiveness and underlying mechanisms. Here, we tested and compared its efficiency on two different tumor models to electrochemotherapy (ECT) and in combination with gene electrotransfer (GET) of a plasmid encoding interleukin-12 (IL-12). We hypothesized that IL-12 potentiates the antitumor effect of local ablative therapies as CaEP and ECT.

**Methods:**

The effect of CaEP was tested *in vitro* as well as *in vivo* in murine melanoma B16-F10 and murine mammary carcinoma 4T1 in comparison to ECT with bleomycin. Specifically, the treatment efficacy of CaEP with increasing calcium concentrations alone or in combination with IL-12 GET in different treatment protocols was investigated. We closely examined the tumor microenvironment by immunofluorescence staining of immune cells, as well as blood vessels and proliferating cells.

**Results:**

*In vitro*, CaEP and ECT with bleomycin reduced cell viability in a dose-dependent manner. We observed no differences in sensitivity between the two cell lines. A dose-dependent response was also observed *in vivo*; however, the efficacy was better in 4T1 tumors than in B16-F10 tumors. In 4T1 tumors, CaEP with 250 mM Ca resulted in more than 30 days of growth delay, which was comparable to ECT with bleomycin. In contrast, adjuvant peritumoral application of IL-12 GET after CaEP prolonged the survival of B16-F10, but not 4T1-bearing mice. Moreover, CaEP with peritumoral IL-12 GET modified tumor immune cell populations and tumor vasculature.

**Conclusions:**

Mice bearing 4T1 tumors responded better to CaEP *in vivo* than mice bearing B16-F10 tumors, even though a similar response was observed *in vitro*. Namely, one of the most important factors might be involvement of the immune system. This was confirmed by the combination of CaEP or ECT with IL-12 GET, which further enhanced antitumor effectiveness. However, the potentiation of CaEP effectiveness was also highly dependent on tumor type; it was more pronounced in poorly immunogenic B16-F10 tumors compared to moderately immunogenic 4T1 tumors.

## 1 Introduction

The involvement of the immune system in the development of tumors as well as in the outcome of treatment is the main focus of cancer research (1). The efficacy of monotherapies, such as chemotherapy or radiotherapy, is often hindered due to the development of resistance to drugs or radiation. Novel treatment strategies use combinatorial therapy that consists of a complementary combination of two or more different therapies, for example, local ablative therapy in combination with immunotherapy. Ablative therapies have a direct cytotoxic effect on tumor cells and have the potential to cause immunogenic cell death by releasing damage-associated patterns (DAMPs), thus priming the immune system to tumor antigens (2–6). However, additional blocking of the negative regulators of immune activation by immune checkpoint inhibitors or stimulating the immune response is also essential in many cases to obtain pronounced antitumor effect (7).

Calcium electroporation (CaEP) is a novel local ablation therapy that arose from the principles of electrochemotherapy (ECT), where supraphysiological concentrations of calcium ions (Ca^2+^) are used to induce cell death instead of toxic cytostatic drugs, such as bleomycin and cisplatin (8–14). As Ca^2+^ is a ubiquitous second messenger involved in many essential cellular processes, its intracellular homeostasis is highly regulated and maintained at low levels (15). When cells are exposed to pulsed electric fields (electroporation (EP)), the cell membrane is transiently permeabilized, allowing the increased entry of Ca^2+^ ions, thus perturbing Ca^2+^ homeostasis and causing cell death (16, 17). This treatment method has already been tested in several clinical trials and veterinary studies on different tumor types and has a similar response rate to ECT with bleomycin (18–24). In addition, it was demonstrated that CaEP can induce high mobility group box 1 (HMGB1) and ATP release indicating immunogenic cell death, therefore it could be used as *in situ* vaccination for combination with immunomodulatory therapies (25).

One of the most prominent and investigated immunostimulators in cancer therapy is interleukin 12 (IL-12), a cytokine that has potent adjuvant activity in cancer, as it utilizes effectors of both innate and adaptive immunity (26). Endogenous IL-12 is mainly produced by activated antigen-presenting cells, such as dendritic cells, monocytes, and macrophages, while its main role is in the induction of proliferation and lytic function of T cells and natural killer (NK) cells. Furthermore, IL-12 stimulates cytokine secretion, especially IFN-γ secretion, which in turn profoundly alters the tumor microenvironment, favoring antitumor phenotype (26, 27). Nevertheless, IFN-γ is also the main mediator of IL-12 systemic toxicity, as seen during clinical testing of recombinant IL-12 (28). Gene electrotransfer (GET) of plasmids encoding IL-12 has been shown to be an effective treatment approach, with controlled release and greater safety, as shown in preclinical settings in mouse models (29–32) and veterinary clinical studies in dogs (33–37). In addition, GET of plasmids encoding IL-12 is currently also in clinical trials, showing promising results (38–40).

In this study, we investigated the effect of CaEP on two different tumor models, murine melanoma B16-F10 and murine mammary carcinoma 4T1. We investigated a difference in treatment efficacy regarding increasing calcium concentrations. Finally, we studied the response of CaEP with GET of plasmids encoding IL-12 in different treatment protocols, which have not yet been elucidated.

## 2 Methods

### 2.1 Cell lines and animals

B16-F10 murine melanoma cells (ATCC) were cultured in Advanced MEM medium (AMEM, Gibco), and 4T1 mammary carcinoma cells were cultured in Advanced RPMI 1640 medium (Gibco). Both media were supplemented with 5% fetal bovine serum (FBS, Gibco), 10 mM L-glutamine (GlutaMAX, Thermo Fisher Scientific), penicillin-streptomycin (100x, Sigma-Aldrich) and held in a 5% CO_2_ humidified atmosphere at 37°C. All cells were routinely tested to be mycoplasma-free (MycoAlert™ Plus Mycoplasma Detection Kit, Lonza).

In experiments, eight- to nine-week-old (18-21 g) female C57Bl/6NCrl (C57Bl/6) and BALB/cAnNCrl (BALB/c) mice (Charles River Laboratories) were housed under specific pathogen-free conditions at a temperature of 20–24°C, a relative humidity of 55 ± 10%, a 12-h light–dark cycle and *ad libitum* food and water. All experimental procedures were performed in compliance with the guidelines for animal experiments of the EU directives (2010/63/EU), ARRIVE Guidelines, and the permission of the Ministry of Agriculture, Forestry, and Food of the Republic of Slovenia (Permission No. U34401-1/2015/43). Mice were randomly assigned to experimental groups (**Table 1**; **Supplementary Table 1**), and the number of animals in each group is indicated in the **Supplementary Tables 3, 4**.

**Table 1:** List and description of treatment groups with 250 mM calcium or ECT/BLM and IL-12 GET.

No.	Group	Treatment
**1**	Control	Injection saline i.t.
**2**	EP	Injection saline i.t. + EP
**3**	IL-12 i.t. + IL-12 p.t.	Injection plasmid solution i.t. and p.t.
**4**	IL-12 i.t. + EP	Injection plasmid solution i.t. + EP
**5**	IL-12 p.t. + GET	Injection plasmid solution p.t. + GET
**6**	IL-12 i.t. + EP + IL-12 p.t. GET	Injection plasmid solution i.t. + EP and injection plasmid solution p.t. + GET
**7**	Ca	Injection 250 mM Ca solution i.t.
**8**	Ca + IL-12 p.t.	Injection 250 mM Ca solution i.t. and plasmid solution p.t.
**9**	Ca + IL-12 p.t. GET	Injection 250 mM Ca solution i.t. and plasmid sollution p.t. + GET
**10**	Ca + IL-12 i.t.	Injection mixture 250 mM Ca solution and plasmid solution i.t.
**11**	Ca + EP	Injection 250 mM Ca solution i.t. + EP
**12**	Ca + EP + IL-12 p.t.	Injection 250 mM Ca solution i.t. + EP and plasmid solution p.t.
**13**	Ca + EP + IL-12 p.t. GET	Injection 250 mM Ca solution i.t. + EP and plasmid solution p.t. + GET
**14**	(Ca + IL-12 i.t.) + EP	Injection mixture 250 mM Ca and plasmid solution i.t. + EP
**15**	(Ca + IL-12 i.t.) + EP + IL-12 p.t.	Injection mixture 250 mM Ca and plasmid solution i.t. + EP AND plasmid solution p.t.
**16**	(Ca + IL-12 i.t.) + EP + IL-12 p.t. GET	Injection mixture 250 mM Ca and plasmid solution i.t. + EP AND plasmid solution p.t. + GET
**17**	BLM	Injection BLM (250 μg/ml) i.t.
**18**	BLM + IL-12 p.t.	Injection BLM (250 μg/ml) i.t. and plasmid solution p.t.
**19**	BLM + IL-12 p.t. GET	Injection BLM (250 μg/ml) i.t. and plasmid solution p.t. + GET
**20**	BLM + IL-12 i.t.	Injection mixture BLM solution and plasmid solution i.t.
**21**	BLM + EP	Injection BLM (250 μg/ml) i.t. + EP
**22**	BLM + EP + IL-12 p.t.	Injection BLM (250 μg/ml) i.t. + EP and plasmid solution p.t.
**23**	BLM + EP + IL-12 p.t. GET	Injection BLM (250 μg/ml) i.t. + EP and plasmid solution p.t. + GET
**24**	(BLM + IL-12 i.t.) + EP	Injection mixture BLM and plasmid solution i.t. + EP
**25**	(BLM + IL-12 i.t.) + EP + IL-12 p.t.	Injection mixture BLM and plasmid solution i.t. + EP AND plasmid solution p.t.
**26**	(BLM + IL-12 i.t.) + EP + IL-12 p.t. GET	Injection mixture BLM and plasmid solution i.t. + EP AND plasmid solution p.t. + GET

### 2.2 Solutions and plasmid

Electroporation buffer (EPB) consisted of 136 mM NaCl, 5 mM KCl, 2 mM MgCl_2_, 10 mM HEPES, and 10 mM glucose (pH 7.2, 300-310 mOsm/kg) (41). The calcium solution was prepared as CaCl_2_×6H_2_O in distilled water to 500 mM in the stock solution. For *in vitro* experiments, calcium solutions diluted in EPB to 5×10^-3^ M, 4×10^-3^ M, 3×10^-3^ M, 2×10^-3^ M, and 1×10^-3^ M concentration in a final cell suspension were used. Bleomycin was diluted in EPB to 1.4×10^-6^ M, 1.4×10^-7^ M, 1.4×10^-8^ M, 1.4×10^-9^ M, and 1.4×10^-10^ M concentration in a final cell suspension. For *in vivo* experiments, 50 mM, 168 mM, and 250 mM calcium solutions diluted in distilled water were used. Bleomycin was diluted in saline solution to a final concentration of 0.177 mM (10 μg/40 μl) for *in vivo* experiments.

The therapeutic plasmid encoding mouse IL-12 (pORF-mIL-12-ORT; pIL-12) (42) was isolated and purified using the EndoFree Plasmid Mega Kit (Qiagen) and diluted in endotoxin-free Milli-Q water. Plasmid quality and concentration were assessed by the 260/280 ratio (Epoch microplate spectrophotometer) and by agarose gel electrophoresis. For the experiments, the plasmid was diluted in saline to a final concentration of 0.25 μg/µl (p.t. application) and 0.5 μg/µl (i.t. application).

### 2.3 *In vitro* electroporation and viability assay

Cells were trypsinized and collected by centrifugation, washed in ice-cold EPB, and prepared for electroporation. Briefly, 50 μl of cell suspension (1×10^6^ cells) served as the treatment control, while the other 50 μl, containing calcium solution or bleomycin (concentrations described in 2.2), was pipetted between two custom-made stainless-steel parallel-plate electrodes (1.9 mm apart), and EP were applied (8 square-wave pulses, 1300 V/cm, 100 μs, 1 Hz) with an electric pulse generator GT-01 (Faculty of Electrical Engineering, University of Ljubljana, Ljubljana, Slovenia). Then, the cells were transferred to a 24-well ultralow attachment plate and incubated for 5 min at room temperature, followed by the addition of 1 ml of culture medium. To determine cell viability after treatment with calcium or bleomycin, alone or in combination with EP, a viability assay with Presto Blue (Thermo Fisher Scientific) was performed. Cells were diluted to 1×10^4^ cells/ml (control groups) or 2×10^4^ cells/ml (EP groups) and transferred to 96-well plates, with each well containing 1×10^3^ cells (exposed to the drug alone) or 2×10^3^ cells (exposed to combined treatment with EP). The plates were then incubated for 72 h in a 5% CO_2_ humidified atmosphere at 37°C. Presto Blue reagent (10 μl/well) was added to cells, followed by 1 h incubation in a humidified incubator at 5% CO_2_ and 37°C. The fluorescence emission was measured using a microplate reader Cytation 1 (BioTek) with a 530/590 nm excitation/emission filter. The measured fluorescence intensity of the treated groups was normalized to the control group or control EP group.

### 2.4 Tumor induction and treatment

**Figure 1:**
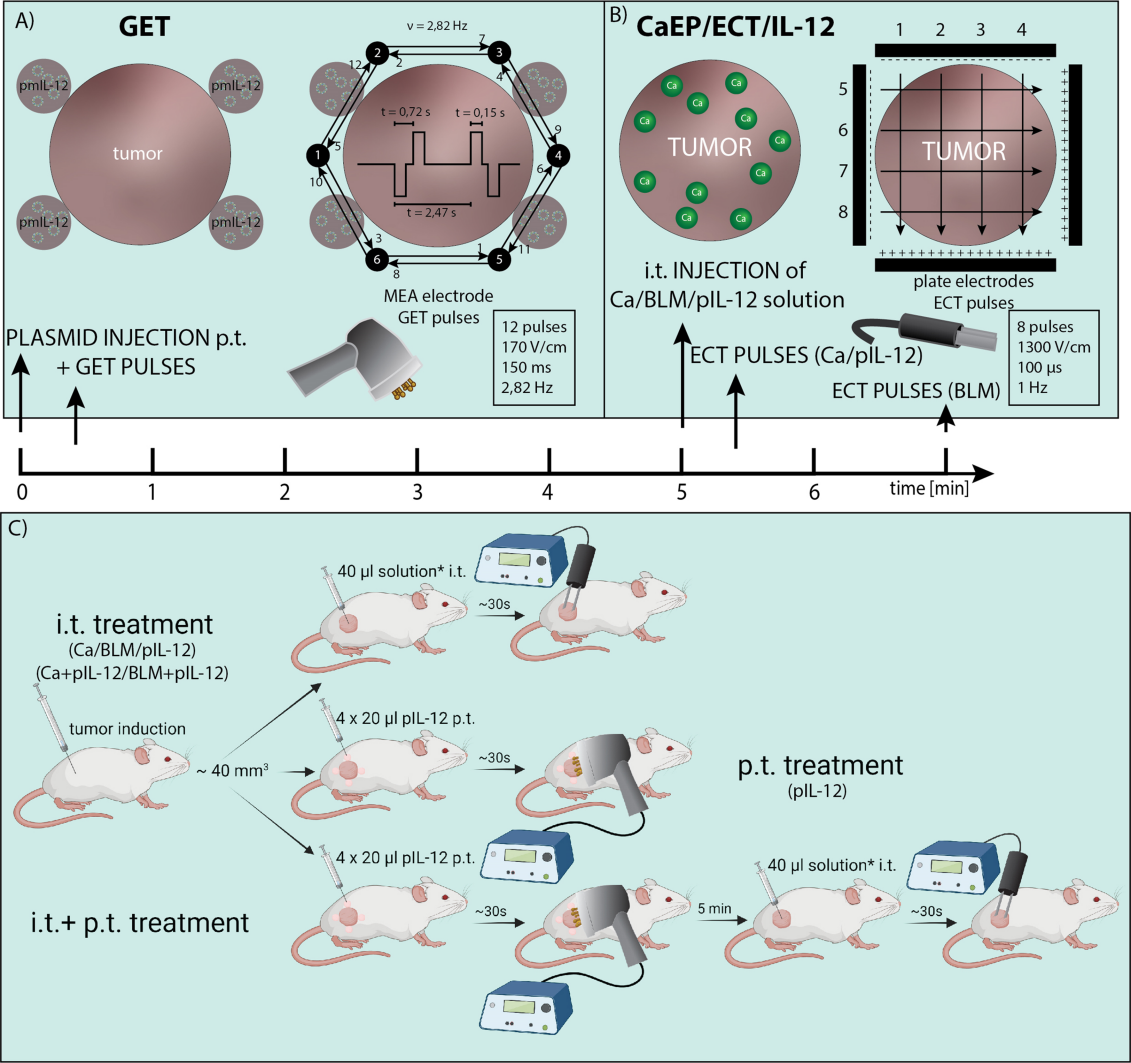
Electric pulses application and treatment protocol. **(A)** Injection site for p.t. GET with non-invasive multi-electrode array (MEA) and distribution of electric pulses between the pins arranged in a circle. **(B)** i.t. injection of calcium, bleomycin and/or pIL-12 and distribution of electric pulses between two parallel stainless-steel electrodes. **(C)** Study design of IL-12 p.t. GET and i.t. CaEP or ECT. The treatment was performed when tumors reached ~40 mm^3^. For i.t. treatment, calcium/bleomycin/pIL-12 or mixture of calcium/bleomycin with pIL-12 was injected prior electroporation (*). For p.t. treatment, pIL-12 was injected in 4 places in tumor proximity prior electroporation.

For induction of tumors, a suspension of 1×10^6^ B16-F10 and 0.5×10^6^ 4T1 cells in 100 μl of 0.9% NaCl was injected subcutaneously into the right flanks of mice. The used tumor models differ in immunogenicity, 4T1 breast carcinoma is more immunogenic than B16-F10 melanoma (43). Treatment was performed when tumors reached volumes of ~40 mm^3^. Throughout the treatment, mice were under inhalation anesthesia with 1.5 – 2.0% isoflurane delivered with anesthesia system connected to oxygen concentrator at a flow rate of 1 l/min (Supera Anesthesia Innovations, Clackamas). The study design and protocol are presented in **Figure 1**.

In the first part, intratumoral (i.t.) therapy was performed with different calcium concentrations (50 mM, 168 mM, and 250 mM in dH_2_O) and bleomycin solution (1.41 mM in saline). Injections were performed with insulin injection needle (29 gauge) and injection time was ~30 sec. In some cases, when injecting 4T1 tumor, due to its high solidity (compactness), the solution was leaking into peritumoral space, regardless of the injection time. EP were delivered immediately after i.t. injection of 40 μl of calcium solution or 2 min after injection of bleomycin. Control group tumors were injected intratumorally (i.t.) with a saline solution. The EP were generated by an ELECTRO cell B10 HVLV (LEROY Biotech, Betatech) with parallel stainless-steel electrodes in 2 sets of 4 pulses in a perpendicular direction (voltage-to-distance ratio 1300 V/cm, frequency 1 Hz, duration 100 μs). Good contact between the skin and electrodes was ensured by using a conductive water-based gel (Ultragel, Budapest, Hungary). The experimental groups are defined in **Supplementary Table 1**.

In the second part, combined treatment consisted of peritumoral (p.t.) IL-12 GET followed by CaEP (250 mM calcium solution) or ECT. Additionally, a plasmid was mixed with either calcium (final concentration 250 mM calcium and 20 μg of plasmid DNA) or bleomycin solution (final concentration 1.4 mM bleomycin and 20 μg of plasmid DNA) immediately before the i.t. injection and subsequent application of EP with the same parameters as above (**Figure 1**). First, p.t. GET was performed using a noninvasive multielectrode array (MEA) with a circular distribution of electrode pins (Iskra Medical). Electric pulses (12 pulses, 170 V/cm, duration 150 ms, frequency 2.82 Hz) were delivered with an electric pulse generator Cliniporator (IGEA) as previously described (43) after p.t. injection of 4×20 μl of pIL-12 (20 μg in total). After 5 min, i.t. treatment was performed. The experimental groups are defined in **Table 1**.

### 2.5 Tumor growth measurements and rechallenge

After the therapy, tumor growth was monitored three times per week by Vernier caliper. The volumes were calculated using the equation V=a×b×c×π/6, where a, b and c represent perpendicular diameters of the tumor. The mice that were tumor-free for 100 days (complete response) were rechallenged with a subcutaneous injection of the same tumor cells in the opposite (left) flank and monitored for tumor outgrowth.

### 2.6 Tumor collection and immunofluorescent staining

On day three after the treatment, three mice from groups 1, 6, 7, 11, 13, and 26 (**Supplementary Table 4**) were sacrificed, and tumors with skin were excised. Tumors were first submerged in 4% paraformaldehyde for 16 h and then incubated in 30% sucrose for 24 h before embedding in OCT medium and snap-frozen in liquid nitrogen. Blocks were stored at -20°C until consecutive frozen sections were cut using a Leica CM1850 cryostat at a thickness of 14 μm in a direction perpendicular to the skin. Slides were dried at 37°C for 10 min, washed for 5 min in phosphate buffered saline (PBS), and then antigen retrieval was performed by immersing the slides in hot citrate buffer (approx. 95°C; 10 mM sodium citrate, 0,05% Tween 20, pH 6.0) and allowed to stand for 1 h at the bench to cool. Slides were then rinsed in two changes of PBS; all subsequent steps were performed in a light-protected humidified chamber. Sections were incubated in permeabilization blocking buffer (5% donkey serum, 0.5% Triton X-100, 300 mM glycine in PBS) for 30 min at room temperature and then rinsed by immersing in PBS for 5 min, followed by 1 h incubation with blocking buffer (5% donkey serum, 300 mM glycine in PBS). Slides were incubated overnight with primary antibodies (**Supplementary Table 2**) in blocking buffer (2% donkey serum, 300 mM glycine in 1x PBS) at 4°C. The next day, the sections were washed 3 times for 10 min with PBS and then incubated with secondary antibodies (**Supplementary Table 2**) for 1 h at room temperature in blocking buffer and washed 3 times with PBS. Nuclear counterstaining was performed by incubation with Hoechst 33342 solution (3 μg/ml in PBS) for 10 min, washed 3 times with PBS, and mounted with Prolong™ Glass Antifade Mountant (Thermo Fisher Scientific).

### 2.7 Microscopy

Three tumor samples per group were imaged with an LSM 800 confocal microscope (Zeiss) with a 20x objective (NA 0.8). Lasers with excitation wavelengths of 405 nm, 488 nm, 561 nm, and 640 nm were used to excite Hoechst 33342, Alexa Fluor 488, Cy3, and Alexa Fluor 647, respectively. For the capture of the emitted light, a gallium arsenide phosphide (GaAsP) detector was used combined with a variable dichroic and filters at channel-specific wavelengths: 410 – 545 nm (Hoechst 33342), 488 – 545 nm (Alexa Fluor 488), 565 – 620 nm (Cy3) and 645 – 700 nm (Alexa Fluor 647). The obtained images were visualized and analyzed with Imaris software (Bitplane). Based on the negative control, cutoff values were determined for each channel.

### 2.8 Statistical analysis

Statistical analysis was performed using GraphPad Prism 9 (GraphPad Software). The significance of viability data was determined by two-way ANOVA with a Holm Sidak multiple comparisons test (vs. control or EP cells). IC_50_ values were calculated by nonlinear regression analysis. Experiments *in vitro* were performed in 3 biological and 8 technical replicates, and data are represented as the means ± SEM unless otherwise stated. The significance of Kaplan Meier survival curves was determined by the Log-rank test. Growth delay was presented with AM ± SE and complete responses were labeled with 100 days of growth delay. The significance of growth delay data was determined using Two-way ANOVA with a Holm Sidak multiple comparisons test. The significance of immunofluorescence data of frozen tissue sections was determined by one-way ANOVA with Dunnett’s multiple comparisons *post hoc* test. The mode of action (additivity, synergism, and antagonism) of treatments was evaluated by the method developed by Spector et al. (44). A P value of <0.05 was considered to be statistically significant (ns P≥0.05; *P 0.01 – 0.05; **P 0.001 – 0.01; ***P 0.0001 – 0.001; ****P <0.0001).

## 3 Results

### 3.1 *In vitro* CaEP and ECT with bleomycin reduced cell viability in a dose-dependent manner

**Figure 2:**
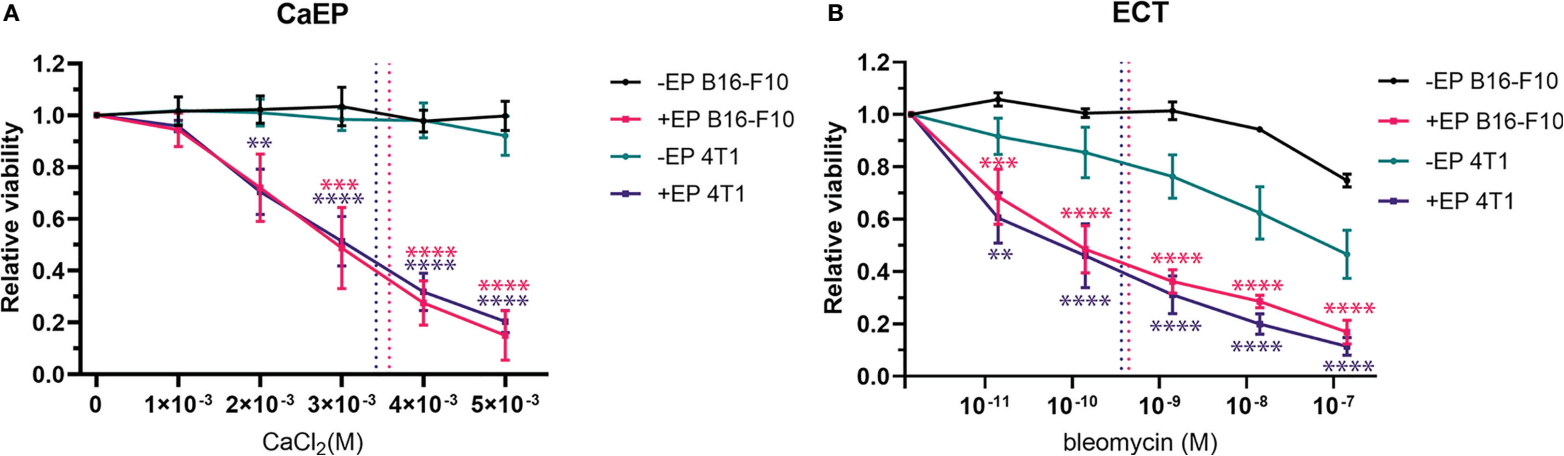
Viability of B16-F10 and 4T1 cell lines after **(A)** CaEP and **(B)** ECT measured after 72 h. Dotted lines represent IC50 values. P value (P < 0.05) is color coded and calculated vs –EP or +EP control cells. The values are presented as AM ± SE. **P 0.001 – 0.01; ***P 0.0001 – 0.001; ****P <0.0001.

The cytotoxicity of CaEP or ECT/BLM on B16-F10 and 4T1 cells was first evaluated to determine the intrinsic sensitivity of the two cell lines to the treatments (**Figure 2**). The exposure of cells to the increased extracellular calcium without EP did not affect the survival of cells (**Figure 2A**). In contrast, exposure to BLM without EP reduced cell survival at the highest concentrations used compared to nontreated cells (**Figure 2B**). When cells were exposed to EP in the presence of calcium, a dose-dependent decrease in cell viability was observed, with IC_50_ values of 3.6×10^-3^ M and 3.4×10^-3^ M calcium for B16-F10 and 4T1 cells, respectively (**Figure 2A**). The same effect was observed after ECT with bleomycin, with IC_50_ values of 4.5×10^-10^ M and 3.7×10^-10^ M bleomycin for B16-F10 and 4T1 cells, respectively (**Figure 2B**). There were no statistically relevant differences between the two cell lines.

### 3.2 Antitumor effectiveness of CaEP with increasing concentrations of calcium

The antitumor effectiveness of CaEP with 50 mM, 168 mM, and 250 mM calcium was tested in B16-F10 melanoma in C57Bl/6 mice and 4T1 mammary carcinoma in Balb/c mice. ECT with bleomycin was used as a positive control.

**Figure 3:**
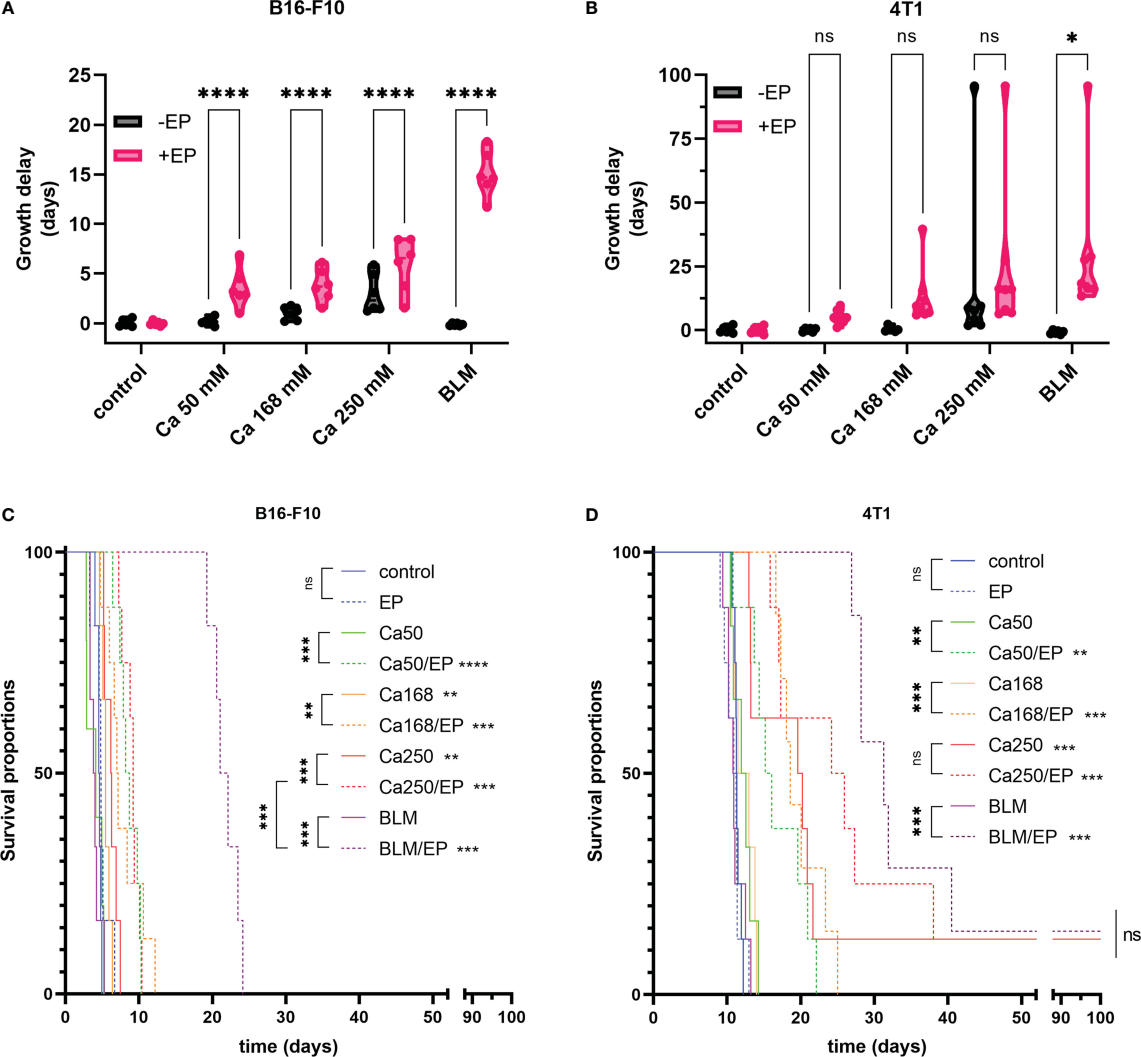
Effectiveness of CaEP or ECT with bleomycin is different in B16-F10 and 4T1 tumors. The response to the therapy is presented by **(A, B)** tumor growth delay and **(C, D)** Kaplan-Meier graphs. In **(A, B)** each dot represents one animal in violin plots showing distribution of data. ns P≥0.05; *P 0.01 – 0.05; **P 0.001 – 0.01; ***P 0.0001 – 0.001; ****P <0.0001.

Treatment of B16-F10 melanoma with CaEP resulted in significantly prolonged survival of animals (P<0.05) in comparison to control or controls with calcium injection alone (**Figure 3C**). The GD of B16-F10 tumors treated with 50 mM, 168 mM, and 250 mM CaEP was 3.6 ± 0.8 days, 3.8 ± 0.7 days, and 5.9 ± 1.1 days, respectively (**Figure 3A**). Injection of 250 mM calcium solution alone resulted in a growth delay of 3.0 ± 0.8 days. The survival of mice treated with ECT with bleomycin was significantly higher, 20.7 ± 1.0 days, compared to Ca/EP (**Figure 3C**; **Supplementary Table 3**).

When 4T1 mammary carcinoma tumors were treated, somewhat different results were obtained. Survival of mice and GD was not much altered after injection of 50 mM and 168 mM calcium alone (**Figure 3D**); however, when 250 mM calcium solution was injected alone, it resulted in significantly prolonged GD of 27.6 ± 12.2 days, whereas after CaEP, it was 32.0 ± 10.0 days. Rather high variability in response to the highest calcium solution could be due to the leakage of the solution due to the injections into the very solid 4T1 tumors. CaEP with 50 mM and 168 mM calcium solutions caused GDs of 5.1 ± 0.9 days and 13.0 ± 4.0 days, respectively. ECT with bleomycin resulted in a GD of 30.9 ± 11.0 days, not significantly higher in comparison to Ca/EP with 250 mM calcium solutions (**Figure 3B, D**; **Supplementary Table 3**).

All treatments were well tolerated by the animals, as no treatment-related mortality or reduced body weight were observed. Morphologically, in both tumor models, edema of the treated area arose in all groups with EP and calcium with or without EP, which resolved until day 3 after the treatment. Concurrently, visible necrosis of tumors developed with the formation of a scab. Nonetheless, most tumors continued to grow from the rim or beneath the scab. Cured Balb/c mice were not resistant to secondary challenge (**Supplementary Table 4**).

### 3.3 Adjuvant p.t. GET of the plasmid encoding IL-12 prolongs the survival of B16-F10-bearing mice after CaEP

The contribution of IL-12 GET to either CaEP or ECT with bleomycin in two different settings was tested in both tumor models, B16-F10 melanoma and 4T1 carcinoma. The concentration of 250 mM calcium solution was chosen for this experiment, where the best response was obtained in the previous experiments evaluating different doses of calcium solutions. Here, IL-12 p.t. GET was performed before intratumoral treatment with CaEP or ECT. Next, the effectiveness of an intratumorally injected solution of mixed pIL-12 and calcium or BLM followed by EP was tested, and finally, IL-12 p.t. GET was added to this setting.

**Figure 4:**
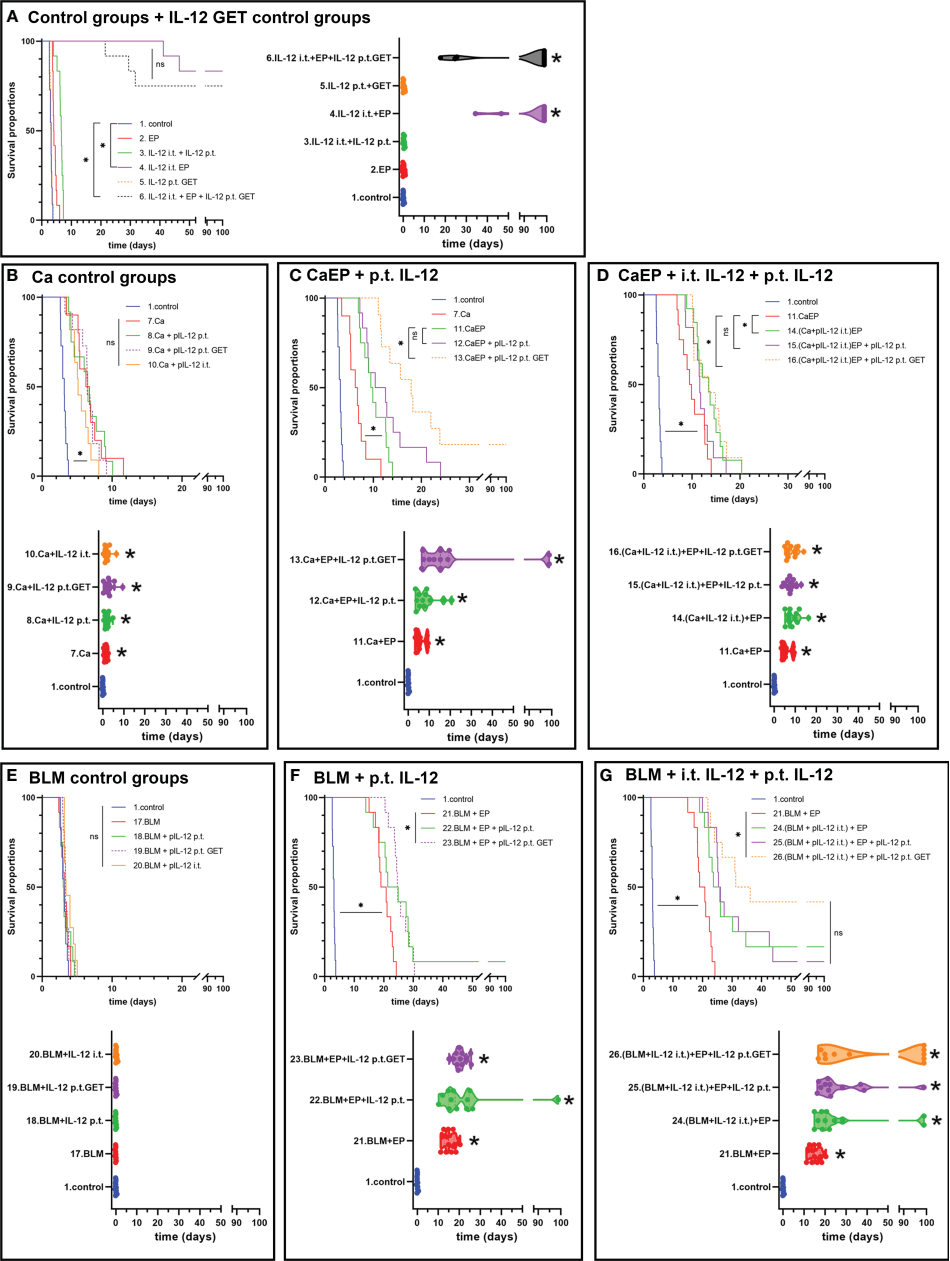
Tumor growth delay and Kaplan-Meier graphs of B16-F10 tumors in response to different treatments. Survival of the animals is significantly prolonged after **(A)** IL-12 i.t. EP alone or in combination with IL-12 p.t. GET. ns P≥0.05; *P<0.05. Adjuvant IL-12 did not significantly contributed to **(B)** Ca injection without electric pulses, but IL-12 p.t. GET significantly prolonged survival of mice after **(C)** CaEP and resulted in tumor cures. **(D)** Combination of i.t. IL-12 and Ca with EP and IL-12 p.t. GET significantly contributed to CaEP with no tumor cures. **(E)** No tumor growth delay was observed after BLM injection, whereas significantly prolonged survival was observed after **(F)** ECT with IL-12 p.t. GET and **(G)** ECT with i.t. IL-12 and IL-12 p.t. GET.

In B16-F10 tumors IL-12 p.t. GET alone did not affect tumor growth. However, IL-12 i.t. EP significantly improved the survival of mice, as up to 83% complete responses were obtained (**Figure 4A**). Similarly, after combinatorial p.t. and i.t. IL-12 GET up to 75% complete responses were achieved despite the double quantity of plasmid being used (**Figure 4A**; **Supplementary Figure 1**, **Supplementary Table 4**). The survival of animals in combinatorial therapy of GET and CaEP or ECT with bleomycin was significantly lower (**Figure 4**). When p.t. GET was performed before injection of calcium alone, some synergistic effect on tumor GD was observed (**Figure 4B**). A significant tumor growth delay of 6.0 ± 0.7 days was obtained after CaEP, which was further potentiated after p.t. GET, where growth delay was 27.0 ± 9.1 days and 18% complete responses were achieved (**Figure 4C**; **Supplementary Table 4**). CaEP with IL-12 i.t. had an additive effect as opposed to CaEP alone, with 8.9 ± 0.9 days of GD; however, IL-12 p.t. GET did not further enhance antitumoral effectiveness (**Figure 4D**). BLM/EP was more effective on B16-F10 tumors than CaEP, with 15.2 ± 0.8 days of tumor GD (**Figure 4F**). An additive effect was observed when IL-12 p.t. was injected before ECT/BLM with 8% complete responses; however, no tumor cures were achieved after IL-12 p.t. GET (**Figure 4F**; **Supplementary Table 4**). When ECT/BLM with IL-12 i.t. was performed, 17% complete responses were obtained, and additional IL-12 p.t. GET resulted in up to 42% tumor cure (**Figure 4G**; **Supplementary Table 4**).

**Figure 5:**
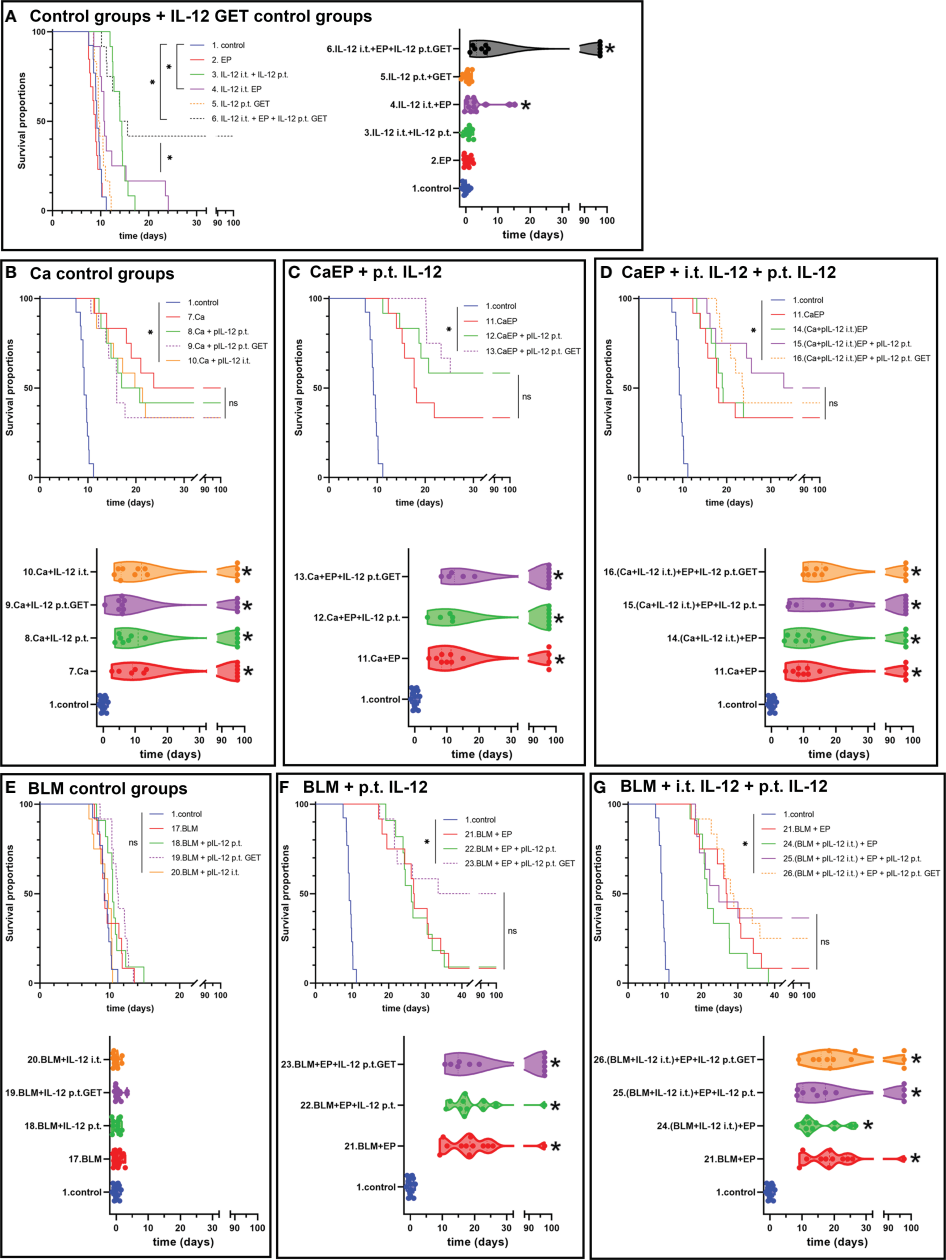
Tumor growth delay and Kaplan-Meier graphs of 4T1 tumors in response to different treatments. Survival of the animals is significantly prolonged after **(A)** IL-12 i.t. EP and i.t. with IL-12 p.t. GET. **(B)** Ca injection resulted in tumor cures, with no contribution of adjuvant IL-12 p.t. GET, also after **(C)** CaEP with IL-12 p.t. GET and **(D)** CaEP with i.t. and p.t. IL-12 GET. ns P≥0.05; *P<0.05 **(E)** No tumor growth delay was observed after BLM injection, whereas significantly prolonged survival was obtained after **(F)** ECT with IL-12 p.t. GET and **(G)** ECT with i.t. IL-12 and IL-12 p.t. GET.

To determine the effect of adjuvant IL-12 GET on 4T1 tumors, which are moderately immunogenic compared to poorly immunogenic B16-F10 tumors (43), the same treatment protocol was used. In this model, IL-12 i.t. GET significantly delayed tumor growth by 4.0 ± 1.5 days, whereas additional IL-12 p.t. GET achieved 42.9 ± 13.8 days of tumor GD and up to 42% complete responses (**Figure 5A**; **Supplementary Figure 2**, **Supplementary Table 4**). In contrast to B16-F10 tumors, calcium injection alone resulted in 50% cures in 4T1 tumors (**Figure 5B**). When EP was added, no further potentiation was observed. Additionally, IL-12 p.t. GET have some additive effect on CaEP alone (**Figures 5C, D**). BLM/EP resulted in 24.2 ± 6.8 days of GD and an 8% survival rate (**Figures 5E, F**; **Supplementary Table 4**). When IL-12 p.t. GET was added to the treatment, efficiency was potentiated to 56.2 ± 12.3 days of GD and 50% tumor cures. BLM/EP with IL-12 i.t. resulted in no complete responses, with 21.4 ± 7.0 days of GD; however, IL-12 p.t. alone or with GET pulses resulted in 36% and 25% complete responses, respectively (**Figure 5G**; **Supplementary Table 4**). Again, cured mice were not resistant to secondary challenge (**Supplementary Table 4**).

### 3.4 CaEP with IL-12 p.t. GET modifies tumor immune cell populations and tumor vasculature

**Figure 6:**
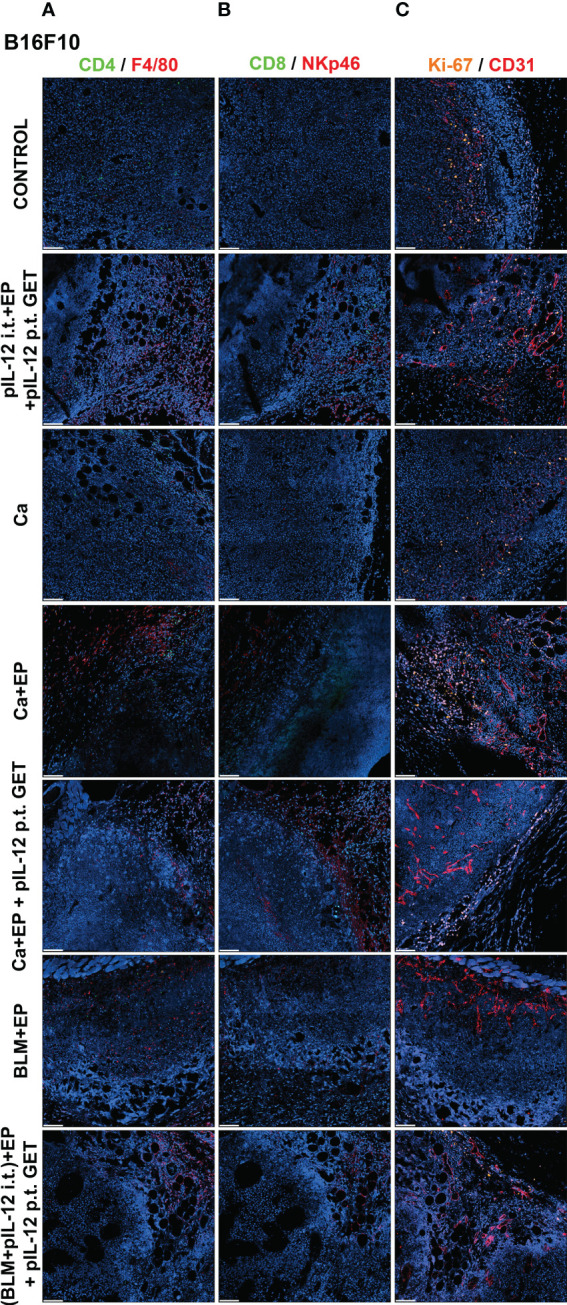
GET of IL-12 impacts infiltration of immune cells to B16-F10 tumors. Tumors were collected on day 3 after treatment for histological analysis. Frozen sections of tumor tissue were stained with **(A)** anti-CD4 (green, Alexa 488) and anti-F4/80 (red, Alexa 647), **(B)** anti-CD8 (green, Alexa 488) and anti-NKp46 (red, Alexa 647) and **(C)** anti-Ki-67 (orange, Cy3) and anti CD31 (red, Alexa 647). Nuclei (blue) were stained with Hoechst 33342. Scale bar: 100 μm.

**Figure 7:**
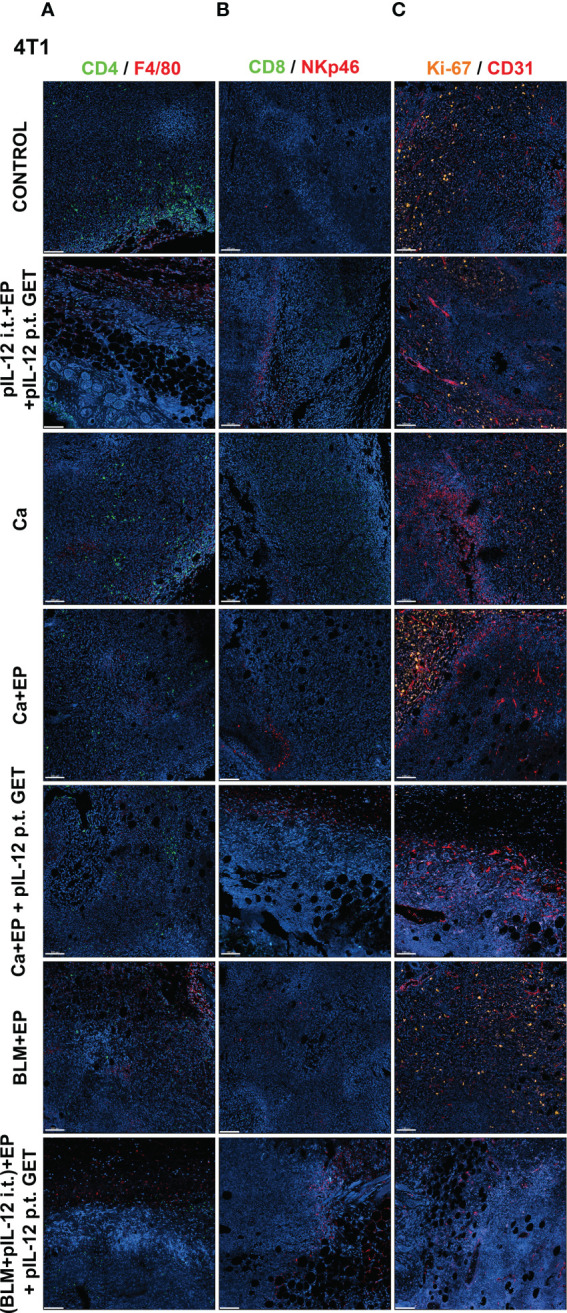
GET of IL-12 impacts infiltration of immune cells to 4T1 tumors. Tumors were colected on day 3 after treatment for histological analysis. Frozen sections of tumor tissue were stained with **(A)** anti-CD4 (green, Alexa 488) and and anti-F4/80 (red, Alexa 647), **(B)** anti-CD8 (green, Alexa 488) and anti-NKp46 (red, Alexa 647) or **(C)** anti-Ki-67 (orange, Cy3) and anti CD31 (red, Alexa 647). Nuclei (blue) were stained with Hoechst 33342. Scale bar: 100 μm.

To determine if GET of a plasmid encoding IL-12 in combination with either CaEP or BLM/EP leads to the infiltration of immune cells in tumors or their proximity, frozen sections of tumors with surrounding skin were excised three days after the therapy and immunofluorescently stained for macrophages (surface expression of F4/80) (**Figures 6A**, **7A**), CD4+ helper T lymphocytes (**Figures 6A**, **7A**), NK cells (surface expression of NKp46) (**Figures 6B**, **7B**), CD8+ T lymphocytes (**Figures 6B**, **7B**), proliferation (Ki-67 nuclear expression) (**Figures 6C**, **7C**) and blood vessels (expression of CD31) (**Figures 6C**, **7C**).

**Figure 8:**
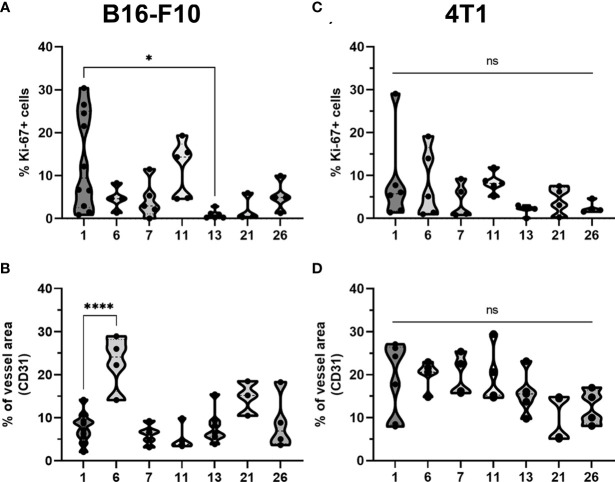
Statistical analysis and graphical presentation of immunfluorescence data from frozen tumor tissue sections. Graphs represent percentage of Ki-67 positive cells and percentage of tumor vessels after different treatments in **(A, B)** B16-F10 and in **(C, D)** 4T1 tumors. The values are presented with violin plots with data distribution. ns P≥0.05; *P 0.01 – 0.05; ****P <0.0001.

The percentage of proliferating cells in the control groups was similar in both tumor models; however, B16-F10 tumors had greater proliferation potential. There was a significant decline in Ki-67+ cells after CaEP with IL-12 p.t. GET (group 13) in the B16-F10 tumor model, but no significant changes were observed in the other groups (**Figures 6C**, **8A**). In 4T1 tumors (**Figure 7C**), there was marginally decreased Ki-67 in tumors after CaEP with IL-12 p.t. GET (group 13) and (BLM+IL-12) i.t. + EP + IL-12 p.t. GET (group 26) (**Figure 8C**). There was an insignificant increase in Ki-67+ cells after CaEP alone, as a pool of highly proliferating cells was located next to the necrotic area (**Figures 6C**, **7C**). This is in accordance with observations of tumor growth after therapy, where tumors continue to grow from under or from the rim of the scab, which forms after treatment due to tumor necrosis.

There was a significant difference between the two tumor models in their vascularization, where the percentage of tumor vessel area was 7.7% for B16-F10 tumors (**Figure 8B**) and 19% for 4T1 tumors (**Figure 8D**). There are also morphological differences in the vasculature of the two tumor models; in B16-F10 tumors, the vessels are sparse and larger in diameter, whereas in 4T1 tumors, they are denser, smaller in diameter, and more evenly arranged (**Figures 6C**, **7C**). In both tumor models, staining of vessels was predominantly in the tumor margin, while very little staining occurred in the tumor core. There was a significant increase in the percentage of vessel area in B16-F10 tumors after treatment with i.t. and p.t. IL-12 GET alone, but no changes after other treatments (**Figure 8B**).

**Figure 9:**
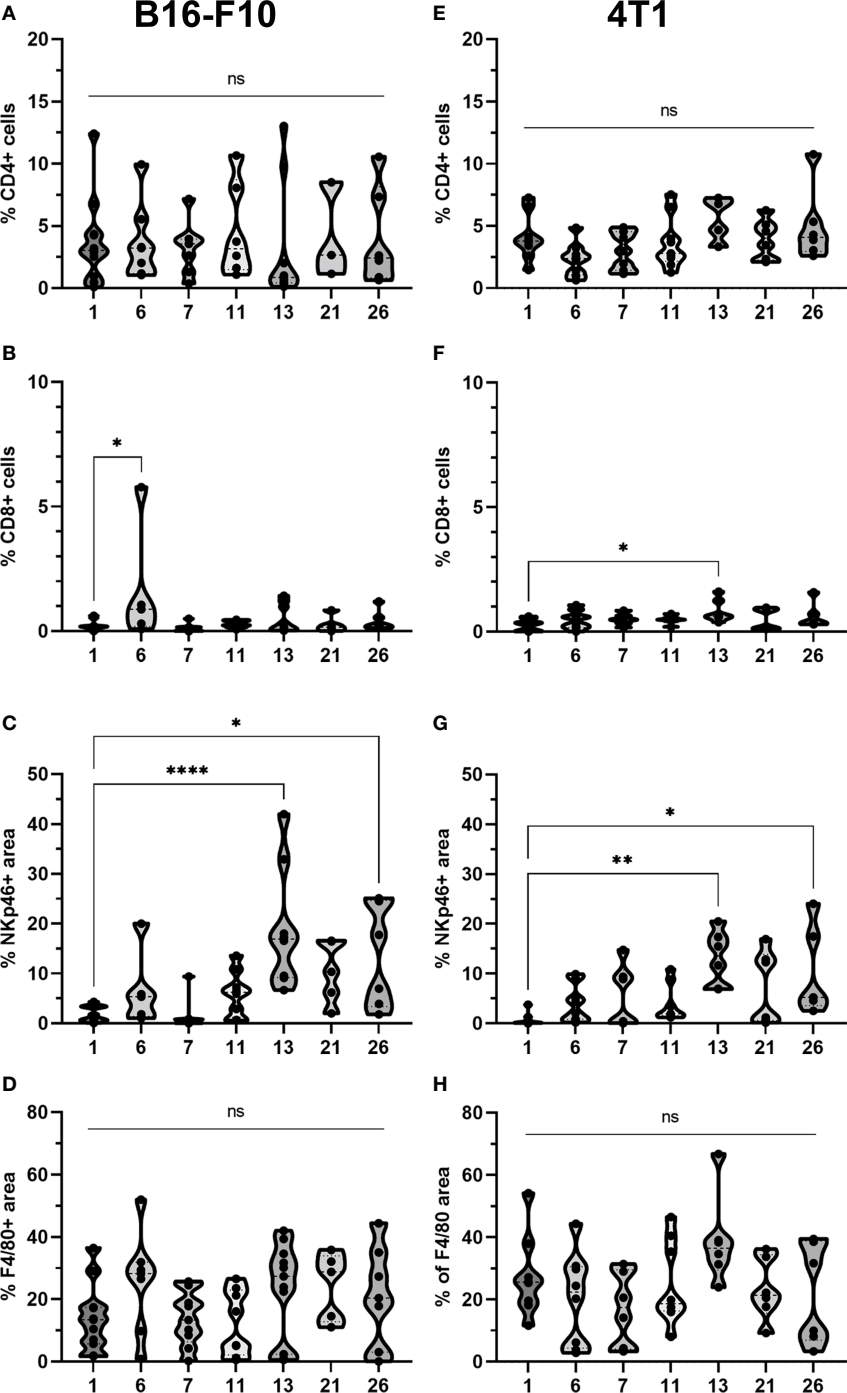
Statistical analysis and graphical presentation of immunfluorescence data from frozen tumor tissue sections. Graphs represent percentage of CD4 and CD8 positive cells, percentage of NKp46 and F4/80 positive area after different treatments in **(A-D)** B16-F10 and in **(E-H)** 4T1 tumors. The values are presented with violin plots with data distribution. ns P≥0.05; *P 0.01 – 0.05; **P 0.001 – 0.01; ****P <0.0001.

The presence of all four stained immune cell populations was confirmed in both B16-F10 (**Figure 6**) and 4T1 tumors (**Figure 7**); however, in control tumors, populations of CD8+ cells and NKp46+ cells were very sparse (**Figures 6B**, **7B**). In both tumor models, the F4/80+ area was mostly present in the tumor edge and the nearby skin, whereas CD4+ cells were predominantly found at the tumor edge (**Figures 6A**, **7A**). After different treatments, there were no significant changes in the F4/80+ area or CD4+ cell count in both tumor models (**Figures 9A, D, E, H**).

In the B16-F10 tumor model, there was a significant increase in the CD8+ cell count after p.t. and i.t. IL-12 GET (group 6) (**Figure 9B**), whereas in the 4T1 model, only after CaEP with IL-12 p.t. GET (**Figure 9F**). The most significant modifications were found when interrogating populations of NK cells, where there was a significant increase in NKp46-positive area in both tumor models after CaEP with p.t. IL-12 GET (group 13) and ECT/BLM with i.t. and p.t. IL-12 GET (group 26) and was concentrated in the proximity of the necrotic area (**Figures 6B**, **7B**, **9C, G**).

## 4 Discussion

CaEP was first described as a potential anticancer treatment in a study published in 2012, where it was shown to be effective in inducing cell death *in vitro* and tumor necrosis *in vivo* (8). After a few preclinical studies that were performed subsequently, the first clinical trial was initiated in 2017 on cutaneous metastases (19), where CaEP and ECT with bleomycin were compared and showed similar effectiveness. In our study, we compared the effectiveness of CaEP with different calcium concentrations alone and in combination with GET of a plasmid encoding IL-12. We demonstrated that the efficacy of CaEP was, regardless compared *in vitro* sensitivity, tumor type dependent; being more effective in 4T1 mammary adenocarcinoma. In contrast, IL-12 i.t. GET alone led to high degree of complete responses in poorly immunogenic B16F10 melanoma tumors. CaEP efficacy can be potentiated by IL-12; however, this was also highly dependent on tumor type; it was more pronounced in poorly immunogenic B16-F10 melanoma compared to moderately immunogenic 4T1 mammary adenocarcinoma tumors.

The mechanism behind CaEP is highly complex, as calcium is a ubiquitous second messenger involved in the regulation of various cellular processes, including cell death (16). High intracellular calcium concentration can activate apoptosis or necrosis, which is dependent on the severity of the stimulus, and it might also cause activation of proteases and lipases and generation of reactive oxygen species, but the most important might be associated with depletion of ATP due to increased consumption to excrete calcium excess and decreased production of ATP due to disturbance in mitochondrial membrane potential (15, 45–48).

In other studies on CaEP *in vitro*, concentrations ranging from 0.5 – 5 mM were tested, demonstrating a wide range of sensitivity across different cell lines; however, normal cells appear to be less sensitive to increased calcium concentrations than cancer cells (9, 11, 49, 50). For *in vivo* studies, much higher calcium concentrations were used, ranging from 50 – 500 mM, and different injected volumes, where a wide range of calcium doses was proven to be effective in tumor eradication (9). In our study, we first tested the intrinsic sensitivity *in vitro* of the two used cell lines, B16-F10 and 4T1, with concentrations ranging between 1 and 5 mM. The two cell lines have been shown to be similarly sensitive to both CaEP and ECT with bleomycin, which we used as a positive control. However, a very different response was observed *in vivo* when tumors were treated with different calcium doses. Injection of 250 mM calcium resulted in minor growth delay in B16-F10 melanoma, and when EP were delivered, this delay was further increased, but not leading to tumor cures. However, in 4T1 carcinoma, some tumors were cured after injection of 250 mM calcium alone, with almost no potentiated effectiveness after delivery of EP. Lower concentrations (50 mM and 168 mM) effectively increased growth delay only when EP were delivered. No significant differences were observed between CaEP with different calcium concentrations, although there was a marginal trend of dose dependency, and 250 mM calcium outperformed lower concentrations. This is in accordance with another study, where they tested different concentrations (100 – 500 mM) and injected volumes (168 mM in a volume equivalent to 20% – 80% of tumor volume) in a tumor model of human breast cancer MDA-MB-231. They showed similar efficacy at all concentrations and volumes used (9). In another study, where they tested CaEP with 168 mM calcium on the tumor model of murine colon carcinoma CT26, 200 μl of calcium solution was injected intratumorally and resulted in 100% tumor cures (14), which is 5 times larger volume as was used in our experiments and 3.4 times larger dose of calcium. This can partly explain the high efficiency of CaEP in that study; however, since rechallenged mice have not developed tumors, the activation of immune system was demonstrated thus making CaEP a local therapy that can be potentially used as *in situ* vaccination (14).

The antitumor effectiveness of CaEP alone has been evaluated in many *in vitro* and *in vivo* studies (8–14, 50–60), as well as in several veterinary (20, 61) and clinical trials (18, 19, 21–24, 62–64), but its efficacy in combination with GET of plasmids encoding IL-12 has not yet been explored. The efficacy of ECT with bleomycin in combination with IL-12 GET has already been described in B16-F10 melanoma and 4T1 carcinoma (43), as well as in CT26 carcinoma, where it was shown that immunogenicity of the tumor model plays a major role in the effectiveness of the therapy. In general, the more immunogenic tumors 4T1 and CT26 responded better to ECT alone than the poorly immunogenic tumors B16-F10, which was also confirmed in our study using ECT with bleomycin as well as also in the case of CaEP, confirming its potential to induce immunogenic cell death. However, IL-12 p.t. GET significantly potentiated the local effectiveness of ECT in B16-F10 tumors, whereas in 4T1 and CT26 tumors, GET of IL-12 did not contribute to the already high antitumor effectiveness of ECT (43). Similar results were obtained in this study, where the contribution of GET of IL-12 to CaEP or ECT with bleomycin was tested after i.t. and p.t. application of IL-12 GET. In this study, suboptimal doses of calcium, bleomycin and IL-12 were used to evaluate the contribution of IL-12 GET. We obtained significant improvement in B57Bl/6 mice survival after CaEP with p.t. IL-12 GET with 18% tumor cure, whereas i.t. application of IL-12 or even i.t. and p.t. application had only a small additive or synergistic effect. Nevertheless, in B16-F10 IL-12 GET mice, either i.t. or i.t. and p.t. combination resulted in the best outcome with up to 83% of tumor cures, which is in compliance with other studies (31, 42, 65, 66) that were also performed on B16-F10 melanoma, where higher doses of IL-12 were used (50 μg), whereas in our study, 20 μg of plasmid was used for i.t. GET and (additional) 20 μg for p.t. GET. Thus, i.t. application of IL-12 in combination with EP in poor immunogenic B16-F10 tumors is probably enough to elicit intratumoral infiltration of CD8+, that led to high tumor curability. Compared to p.t. application, it seems that it is crucial that tumor cells also produce IL-12 for this pronounced antitumor effect.

Different responses to the treatment of IL-12 treatment alone were observed in the moderately immunogenic 4T1 tumor model, where i.t. GET of IL-12 prolonged survival of mice, but did not lead to tumor cures. However, combined p.t. and i.t. GET of IL-12 resulted in 42% of tumor cures, indicating that higher amount of IL-12 is needed for better antitumor effectiveness in this tumor model, however further studies are needed to pin the underlying mechanisms for the obtained results, as there were no differences between the treatment groups regarding the infiltration of immune cells, proliferation status or blood vessels’ area. With additional injection of 250 mM calcium alone to IL-12, up to 50% complete responses were obtained, with no improvement in survival after either EP or GET. After ECT with bleomycin, there was a significant contribution of IL-12 GET to the prolonged survival of animals. One of the important factors affecting the outcome of the combination treatment of CaEP and IL-12 GET may be the immunogenicity of the tumors, as is the case after ECT, where more immunogenic tumors respond better to the treatment (43). Similarly, IL-12 GET has little or no effect in combination with either CaEP or ECT if the monotherapy already has a high response rate. In veterinary studies on spontaneous canine oral malignant melanoma (33) and mast cell tumors (35), immunostimulation with IL-12 p.t. GET potentiated the effectiveness of ECT with either bleomycin or cisplatin on treated tumors and proved to be safe and well tolerated. Additionally, when i.t. ECT and IL-12 GET were performed on canine mast cell tumors, a much better response to the treatment was obtained than with IL-12 p.t. GET resulted in prolonged disease- and progression-free intervals (37). In other studies (67–69), various spontaneous canine tumors were treated with a combination of ECT with IL-12 GET, with plasmid and chemotherapeutic drug injected concurrently and resulted in a good antitumor response in several different tumor types, except sarcoma. In our study, in B16-F10 melanoma, the combination of i.t. ECT and IL-12 GET resulted in 17% complete responses when combined with additional IL-12 p.t. GET, 42% complete responses were reached (**Figure 4**; **Supplementary Table 4**). These results were in conflict with our expectations, as we expected that IL-12 would potentiate the effect of ECT or CaEp; however, both local ablative therapies worsened the antitumor effect obtained by i.t. GET of IL-12 in B16-F10, where 83% of tumor cures were obtained. Most probable reason for this could be the low dose of plasmid DNA used in our study (20 μg), which could be degraded by addition of bleomycin or calcium solution in the combined treatment protocols and thus the expression of IL-12 was not high enough to induce the immunostimulatory effect. Namely, in other studies, where potentiation of the effect was observed higher doses of plasmid DNA were used (up to 2 mg/tumor in canine studies) (35, 45, 67–69). In addition, CaEP was successfully used for termination of transgene expression in muscle (70). This speculation is further supported by the fact, that in both, B16-F10 melanoma and 4T1 breast carcinoma, the antitumor effect of local ablative therapies was more potentiated by IL-12 p.t. GET. Specifically, 4T1 breast carcinoma responded better to ECT with IL-12 p.t. GET, as it resulted in 50% complete responses, whereas the combination of ECT with only IL-12 i.t. GET did not result in tumor cure (**Figure 5**; **Supplementary Table 4**).

The immune system plays an important role in the elimination of tumors. In our study, we closely examined the tumor microenvironment by immunofluorescence staining of immune cells, such as CD4+ helper T lymphocytes, CD8+ T lymphocytes, NK cells and F4/80 macrophages, as well as presence and distribution of blood vessels and Ki-67+ proliferating cells. We did not observe any significant modifications in CD4+ and macrophage populations after the treatments, whereas a higher count of CD8+ cells was observed after i.t. and p.t. IL-12 GET in B16-F10 melanoma, and after CaEP with p.t. IL-12 GET in 4T1 breast carcinoma (**Figures 6**, **7**). Most significant changes were observed in NK cell populations, which significantly increased after combinatorial therapies with IL-12 GET with either CaEP or ECT in both tumor models (**Figure 9**). This implies that our therapy mostly induced the innate immune system, which might be the reason for tumor formation after the rechallenge, indicating that no immune memory was formed. However, since the tumors were excised at day 3, an increase in different immune cell populations, specifically the cells of the adaptive immune response, might be observed at later time points. In one study, high infiltration of M1 macrophages was observed 8 days after IL-12 i.t. GET in B16-F10 melanoma (42). Another study showed high infiltration of granzyme B-positive cells on days 4 and 6 (43) after ECT with IL-12 p.t. GET in B16-F10 melanoma and 4T1 breast carcinoma, but none of the cured mice were resistant to secondary challenge, as was also the case in our study.

A limitation in our study was that only one dose of calcium was tested in combination treatments, which proved to be the most effective in B16-F10 melanoma, whereas in 4T1 breast carcinoma, a lower concentration might prove to be more susceptible to potentiation with IL-12 GET. In addition, to elucidate the immunological mechanisms of the therapy, other time points should be included in immunohistochemical staining of immune cells.

To conclude, this study shows that tumors responded to CaEP in a different way *in vivo*, even though a similar response was observed *in vitro*. This indicates that other, non-direct mechanisms are involved in tumor elimination, and one of the most important factors might be involvement of the immune system. This was confirmed with the combination of CaEP or ECT with IL-12 GET, which further enhances the antitumor effectiveness of the therapy; however, this effect was highly dependent on the tumor immunogenicity and effectiveness of CaEP or ECT alone. Although tumor cures were obtained after the therapy, no immune memory was formed, which may be predominantly due to induction of the innate immune response. Optimizing the calcium concentration and plasmid dosing regimen as well as the timing of GET could still result in different responses, thus further studies are warranted. Also with regard to elucidating antitumor mechanism associated with the tumor type specificity to CaEP and combinational therapies.

## Data availability statement

The raw data supporting the conclusions of this article will be made available by the authors, without undue reservation.

## Ethics statement

The animal study was reviewed and approved by National Ethics Committee for Experiments on Laboratory Animals of Administration of the Republic of Slovenia for Food Safety, Veterinary Sector and Plant Protection of the Ministry of Agriculture, Forestry and Food of the Republic of Slovenia (Permission No. U34401-1/2015/43).

## Author contributions

Conceptualization: MC, BL. Methodology, data acquisition and analysis: BL, MC, KUV, BM. Writing: BL. Review and editing: MC, GS, KUV, BM. All authors contributed to the article and approved the submitted version.
