# Supplementary material for: The effectiveness of calcium electroporation combined with gene electrotransfer of a plasmid encoding IL-12 is tumor type-dependent
Source: Front Immunol. 2023 May 25;14:1189960. doi: 10.3389/fimmu.2023.1189960 (PMC10247961; doi:10.3389/fimmu.2023.1189960)
Supplement: Supplementary file 1 [file DataSheet_1.docx]

Supplementary Material

The effectiveness of calcium electroporation combined with gene electrotransfer of a plasmid encoding IL-12 is tumor type-dependent

**Barbara Staresinic^1^, Bostjan Markelc^1,2^, Katja Ursic Valentinuzzi^1,3^, Gregor Sersa^1,2^, Maja Cemazar^1,4*^**

^1^Department of Experimental Oncology, Institute of Oncology Ljubljana, Ljubljana, Slovenia

^2^Faculty of Health Sciences, University of Ljubljana, Zdravstvena pot 5, Ljubljana, Slovenia

^3^Biotechnical Faculty, University of Ljubljana, Jamnikarjeva 101, 1000 Ljubljana

^4^Faculty of Health Sciences, University of Primorska, Polje 42, Izola, Slovenia

***Correspondence:**Maja Čemažar, PhD

mcemazar@onko-i.si


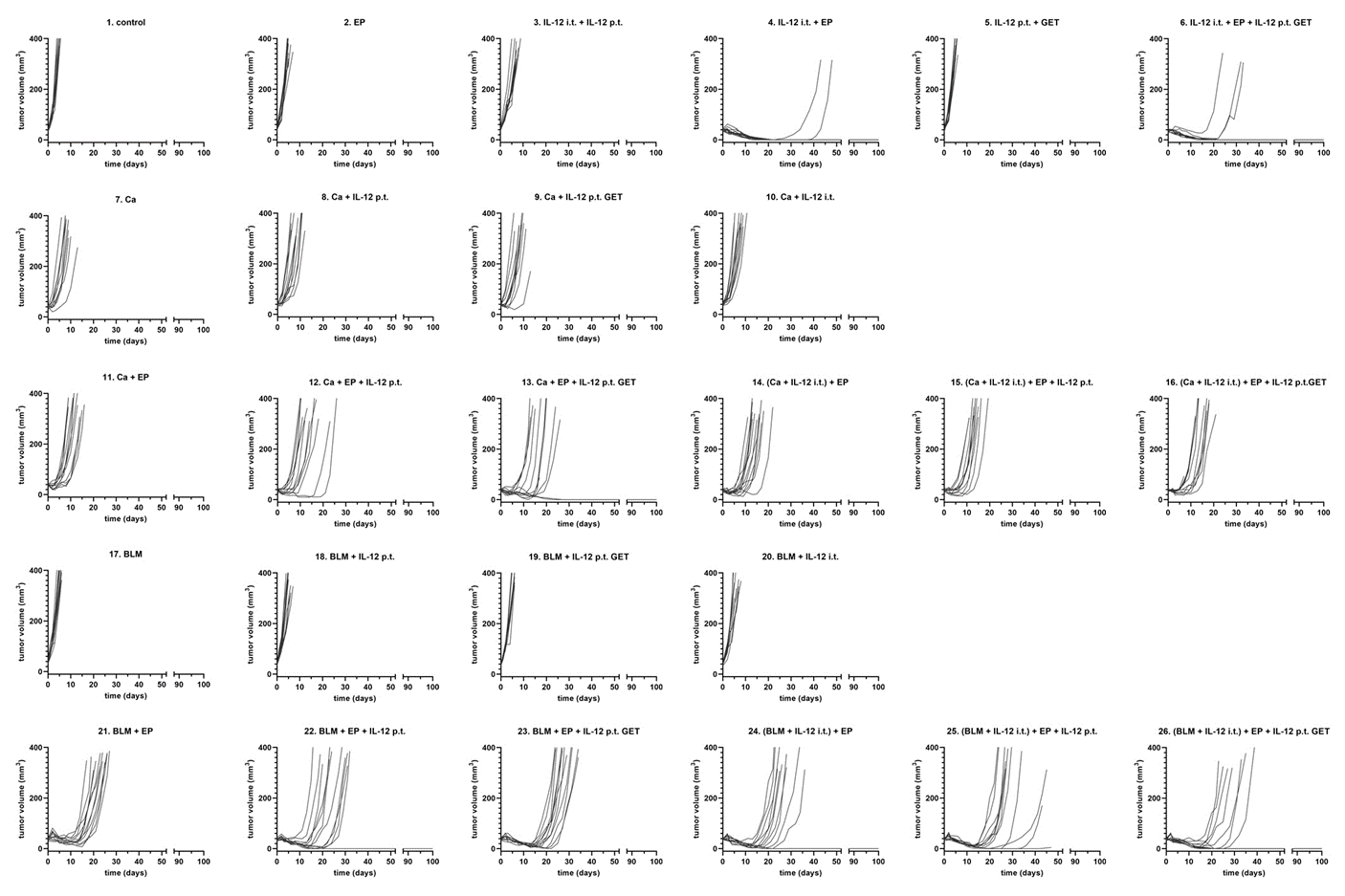
**Supplementary Figure 1**. Growth of B16-F10 tumors after CaEP or ECT/BLM or combination therapy. BLM: bleomycin, Ca: calcium, EP: electroporation, ECT: electrochemotherapy, IL-12: interleukin-12, i.t.: intratumoral, p.t.: peritumoral


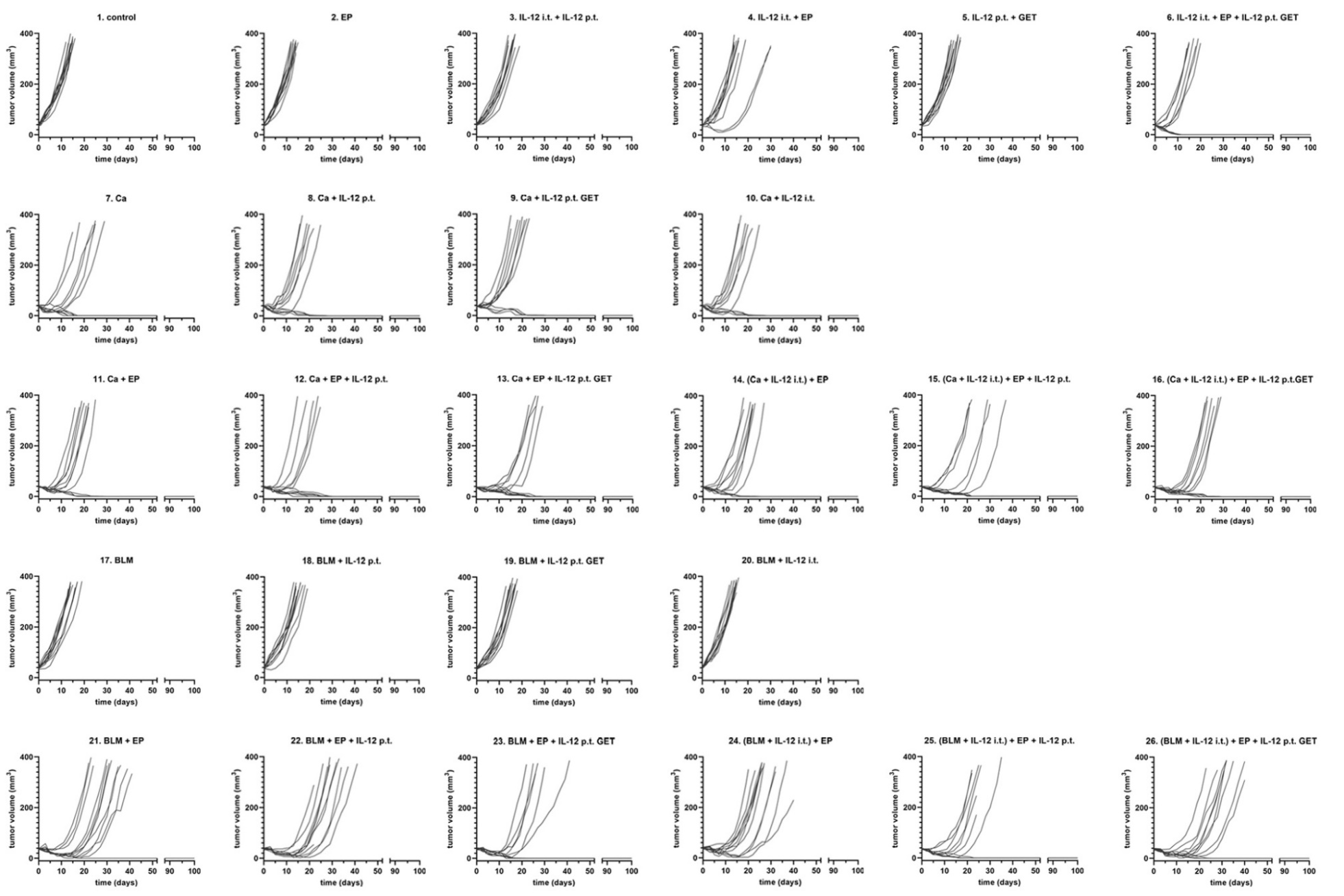
**Supplementary Figure 2.** Growth of 4T1 tumors after CaEP or ECT/BLM or combination therapy. BLM: bleomycin, Ca: calcium, EP: electroporation, ECT: electrochemotherapy, IL-12: interleukin-12, i.t.: intratumoral, p.t.: peritumoral

**Supplementary Table 1**. List and description of treatment groups with different calcium doses and bleomycin.

| **Group** | **Treatment (i.t.)** |
| --- | --- |
| **Control** | Injection saline |
| **EP** | Injection saline + EP |
| **Ca50** | Injection 50 mM Ca solution |
| **Ca50 + EP** | Injection 50 mM Ca solution + EP |
| **Ca168** | Injection 168 mM Ca solution |
| **Ca168 + EP** | Injection 168 mM Ca solution + EP |
| **Ca250** | Injection 250 mM Ca solution |
| **Ca250 + EP** | Injection 250 mM Ca solution + EP |
| **BLM** | Injection bleomycin (250 μg/ml) |
| **BLM + EP** | Injection bleomycin (250 μg/ml) + EP |

**Supplementary Table 2.** List of primary and secondary antibodies used. Listed are antibodies used for immunofluorescence frozen tumor tissue staining. Antibody name, host, dilutions, manufacturer and ID product codes are presented.

| **Primary antibody** | **Host** | **Dilution** | **Manufacturer, catalog num.** |
| --- | --- | --- | --- |
| **anti-CD31/PECAM-1** | goat | 1:200 | R&D Systems, #AF3628 |
| **anti-Ki67** | rabbit | 1:200 | Thermo Fisher Scientific, #RM9106S1 |
| **anti-CD4** | rabbit | 1:200 | Abcam, #ab183685 |
| **anti-CD8-α** | rabbit | 1:200 | Abcam, #ab209775 |
| **anti-F4/80** | rat | 1:200 | Thermo Fisher Scientific, #14-4801-82 |
| **Anti-NKp46** | goat | 1:200 | Thermo Fisher Scientific, #PA5-46986 |
| **Secondary antibody** |  |  |  |
| **Anti-rabbit IgG Alexa Fluor® 488** | goat | 1:500 | Abcam, #ab150077 |
| **Anti-rat IgG Alexa Fluor® 647** | donkey | 1:500 | Jackson Immunoresearch, #712-605-150 |
| **Anti-goat IgG Alexa Fluor® 647** | donkey | 1:500 | Jackson Immunoresearch, #705-605-147 |
| **Anti-rabbit IgG Cy3** | donkey | 1:500 | Jackson Immunoresearch, #711-165-152 |

**Supplementary Table 3.** Average survival time (when tumors reached 300 mm3) and growth delay (GD) is prolonged after CaEP with different calcium doses and ECT/BLM. Data are presented as AM ± SE, data are compared to control group (* P<0.05).

| Group | B16-F10 | | | 4T1 | | |
| --- | --- | --- | --- | --- | --- | --- |
|  | Average survival (days) | GD (days) | N | Average survival (days) | GD (days) | N |
| Control | 4.2±0.1 |  | 6 | 9.8±0.2 |  | 6 |
| EP | 4.1±0.3 | -0.04±0.1 | 6 | 9.9±0.5 | -0.1±0.5 | 6 |
| Ca50 | 3.4±0.4 | 0.2±0.2 | 6 | 10.5±0.6 | 0.1±0.3 | 6 |
| Ca50 + EP | 8.0±0.5* | 3.6±0.8 | 6 | 14.9±1.3 | 5.1±0.9 | 8 |
| Ca168 | 4.6±0.2 | 1.0±0.3 | 6 | 10.8±0.7 | 0.4±0.4 | 6 |
| Ca168 + EP | 6.5±1.0 | 3.8±0.7 | 6 | 18.4±1.2 | 13.0±4.0 | 7 |
| Ca250 | 5.6±0.4 | 3.0±0.8 | 6 | 27.6±12.2* | 18.5±12.9 | 7 |
| Ca25 + EP | 8.5±0.4* | 5.9±1.1 | 6 | 32.0±10.0* | 24.0±10.5 | 8 |
| BLM | 3.3±0.2 | -0.1±0.1 | 6 | 9.2±0.4 | -0.8±0.3 | 6 |
| BLM + EP | 20.7±1.0* | 15.1±0.9 | 6 | 40.4±2.4* | 30.9±11.0 | 7 |

**Supplementary Table 4.** Prolonged survival time (when tumors reached 300 mm^3^) and growth delay (GD) after CaEP or ECT/BLM in combination with pIL-12 GET. Survival time of treatment groups is presented as AM ± SE, data are compared to control group (* P < 0.05).

| **GROUP** | | **B16-F10** | | | | | **4T1** | | | | |
| --- | --- | --- | --- | --- | --- | --- | --- | --- | --- | --- | --- |
|  | | **Survival time (days)** | **GD (days)** | **N** | **CR (%)** | **Secondary resistance (%)** | **Survival time (days)** | **GD (days)** | **N** | **CR (%)** | **Secondary resistance (%)** |
| 1 | Control | 4.1±0.1 | 0±0.1 | 11 |  |  | 12.6±0.3 | 0±0.2 | 13 |  |  |
| 2 | EP | 4.4±0.2 | 0.1±0.2 | 12 |  |  | 12.0±0.3 | 0.4±0.3 | 13 |  |  |
| 3 | IL-12 i.t. + IL-12 p.t. | 6.4±0.3 | 0.3±0.1 | 12 |  |  | 14.1±0.4 | 0.6±0.3 | 12 |  |  |
| 4 | IL-12 i.t. + EP | 90.9±6.2 | 89.1±6.6* | 12 | 83 | 0 | 16.0±1.7 | 4.0±1.5* | 12 |  |  |
| 5 | IL-12 p.t. + GET | 4.3±0.1 | 0.1±0.1 | 12 |  |  | 13.1±0.4 | 0.4±0.3 | 12 |  |  |
| 6 | IL-12 i.t. + EP + IL-12 p.t. GET | 82.3±9.3 | 79.8±10.0* | 12 | 75 | 0 | 50.6±12.6 | 42.9±13.8* | 12 | 42 | 0 |
| 7 | Ca | 8.1±0.8 | 1.5±0.2* | 10 |  |  | 60.4±12.0 | 52.8±13.3* | 12 | 50 | 0 |
| 8 | Ca + IL-12 p.t. | 7.8±0.6 | 2.0±0.4* | 12 |  |  | 52.1±12.2 | 44.5±13.4* | 12 | 42 | 0 |
| 9 | Ca + IL-12 p.t. GET | 8.2±0.6 | 3.1±0.7* | 12 |  |  | 44.8±11.8 | 35.7±13.0* | 12 | 33 | 0 |
| 10 | Ca + IL-12 i.t. | 6.8±0.5 | 2,1±0.5* | 11 |  |  | 46.5±11.5 | 37.9±12.6* | 12 | 33 | 0 |
| 11 | Ca + EP | 11.4±0.7 | 6.0±0.7* | 12 |  |  | 66.4±12.0 | 38.6±12.5* | 12 | 33 | 0 |
| 12 | Ca + EP + IL-12 p.t. | 14.2±1.5 | 8.4±1.6* | 12 |  |  | 68.4±11.3 | 60.4±13.0* | 12 | 58 | 0 |
| 13 | Ca + EP + IL-12 p.t. GET | 32.5±9.3 | 27.0±9.1* | 11 | 18 | 0 | 46.8±11.4 | 61.8±12.5* | 12 | 58 | 0 |
| 14 | (Ca + IL-12 i.t.) + EP | 16.3±1.8 | 8.9±0.9* | 13 |  |  | 62.4±11.4 | 38.6±12.5* | 12 | 33 | 0 |
| 15 | (Ca + IL-12 i.t.) + EP + IL-12 p.t. | 20.5±7.3 | 7.6±0.7* | 11 |  |  | 55.2±11.4 | 54.9±12.7* | 12 | 50 | 0 |
| 16 | (Ca + IL-12 i.t.) + EP + IL-12 p.t. GET | 15.5±1.1 | 8.3±0.8* | 11 |  |  | 13.2±0.5 | 43.6±12.8* | 12 | 42 | 0 |
| 17 | BLM | 6.0±1.7 | -0.1±0.1 | 12 |  |  | 13.8±0.6 | 0.6±0.4 | 12 |  |  |
| 18 | BLM + IL-12 p.t. | 11.8±7.4 | -0.1±0.1 | 12 |  |  | 14.2±0.4 | 0.1±0.4 | 11 |  |  |
| 19 | BLM + IL-12 p.t. GET | 6.3±.15 | 0.0±0.1 | 11 |  |  | 12.5±0.3 | 0.8±0.4 | 12 |  |  |
| 20 | BLM + IL-12 i.t. | 5.2±0.3 | 0.2±0.1 | 11 |  |  | 35.1±6.2 | 0.0±0.3 | 12 |  |  |
| 21 | BLM + EP | 28.0±6.0 | 15.2±0.8* | 12 |  |  | 36.1±6.6 | 24.2±6.8* | 12 | 8 | 0 |
| 22 | BLM + EP + IL-12 p.t. | 36.0±8.0 | 25.1±6.9* | 12 | 8 | 0 | 63.4±11.1 | 25.1±7.3* | 11 | 9 | 0 |
| 23 | BLM + EP + IL-12 p.t. GET | 32.7±5.7 | 20.6±0.9* | 12 |  |  | 25.7±1.4 | 56.2±12.3* | 12 | 50 | 0 |
| 24 | (BLM + IL-12 i.t.) + EP | 37.9±7.7 | 33.1±8.9* | 12 | 17 | 0 | 52.3±11.4 | 21.4±7.0* | 11 |  |  |
| 25 | (BLM + IL-12 i.t.) + EP + IL-12 p.t. | 35.4±5.7 | 30.2±6.6* | 12 | 8 | 0 | 52.3±11.4 | 43.7±12.8* | 11 | 36 | 0 |
| 26 | (BLM + IL-12 i.t.) + EP + IL-12 p.t. GET | 58.7±10.6 | 56.8±11.4* | 12 | 42 | 0 | 47.9±9.2 | 37.9±10.4* | 12 | 25 | 0 |
